# Supplementary material for: GFI1-Dependent Repression of SGPP1 Increases Multiple Myeloma Cell Survival
Source: Cancers (Basel). 2022 Feb 2;14(3):772. doi: 10.3390/cancers14030772 (PMC8833953; doi:10.3390/cancers14030772)

Figure S6: Original western blot images (uncropped blots) showing all the bands with all molecular weight markers on the Western blots from the data presented in the manuscript figures and densitometry readings/intensity ratios.

Figure 1B

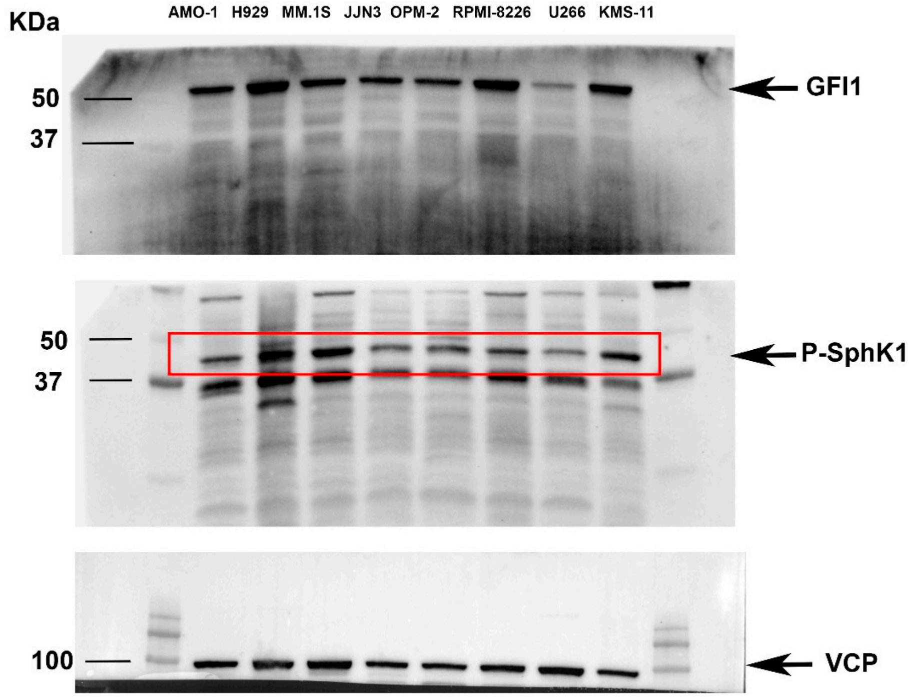

Figure 2 A (insert)

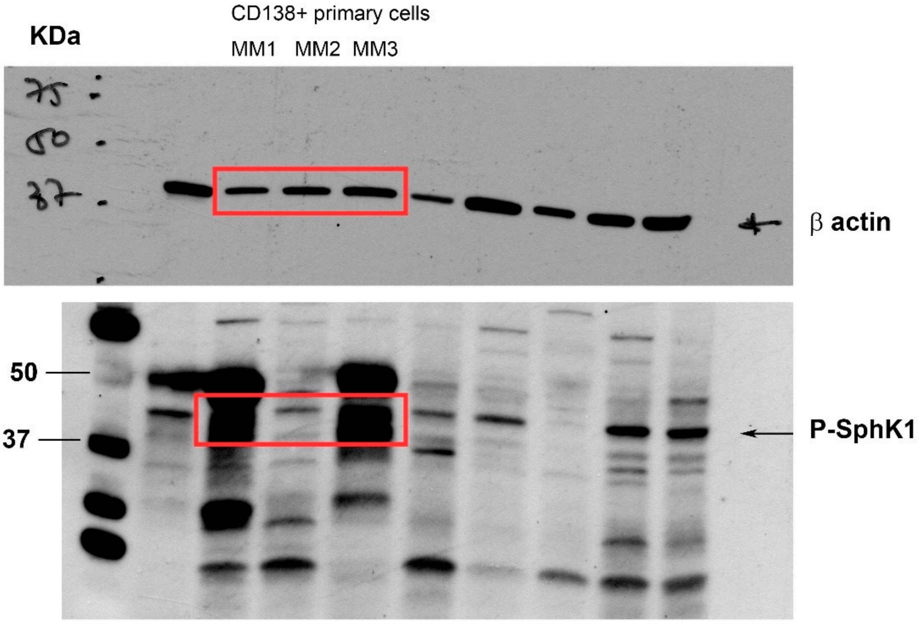

Figure 3 C

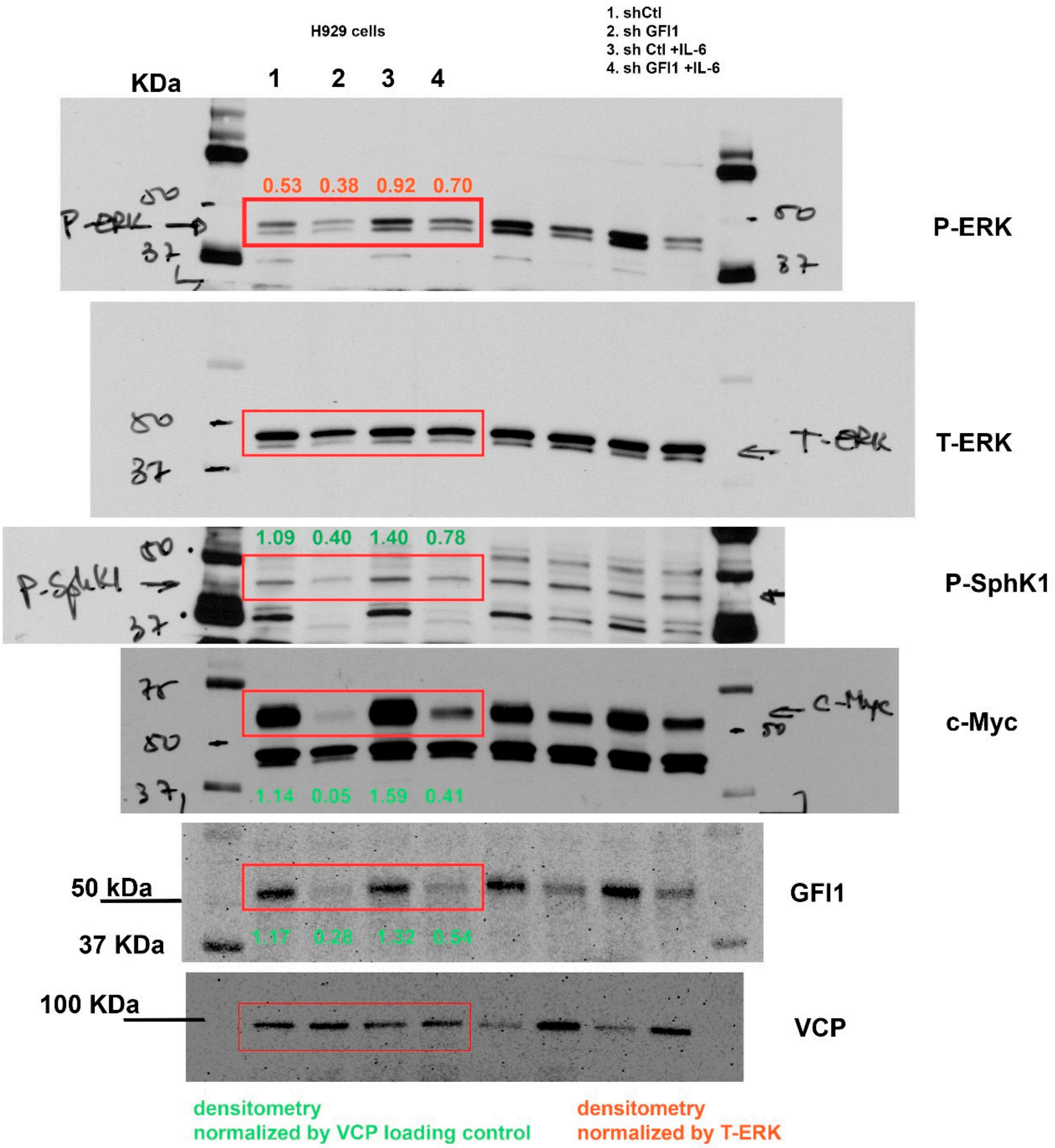
H929 cells
1. shCtl
2. sh GFI1
3. sh Ctl +IL-6
4. sh GFI1 +IL-6
KDa
1 2 3 4
0.53 0.38 0.92 0.70
P-ERK
T-ERK
1.09 0.40 1.40 0.78
P-SphK1
c-Myc
1.14 0.05 1.59 0.41
50 kDa
37 KDa
GFI1
1.17 0.28 1.32 0.54
100 KDa
VCP
densitometry
normalized by VCP loading control
densitometry
normalized by T-ERK

Figure 3 D

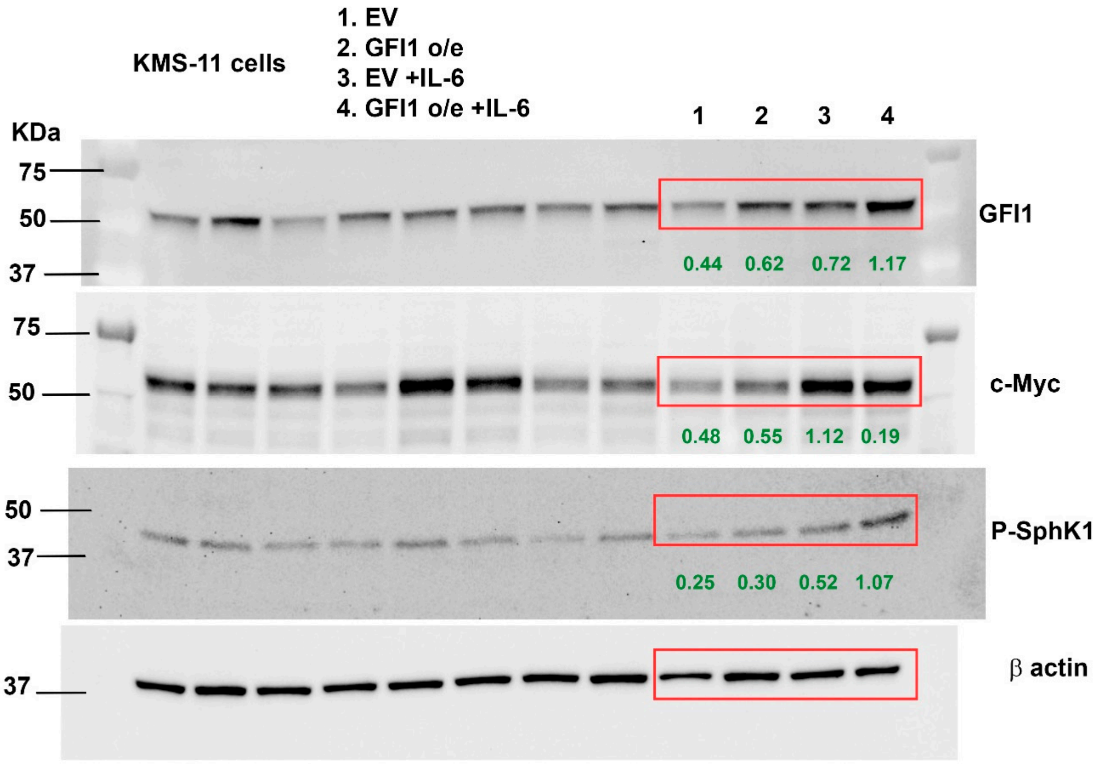

densitometry normalized by β actin loading control

Figure 3 F

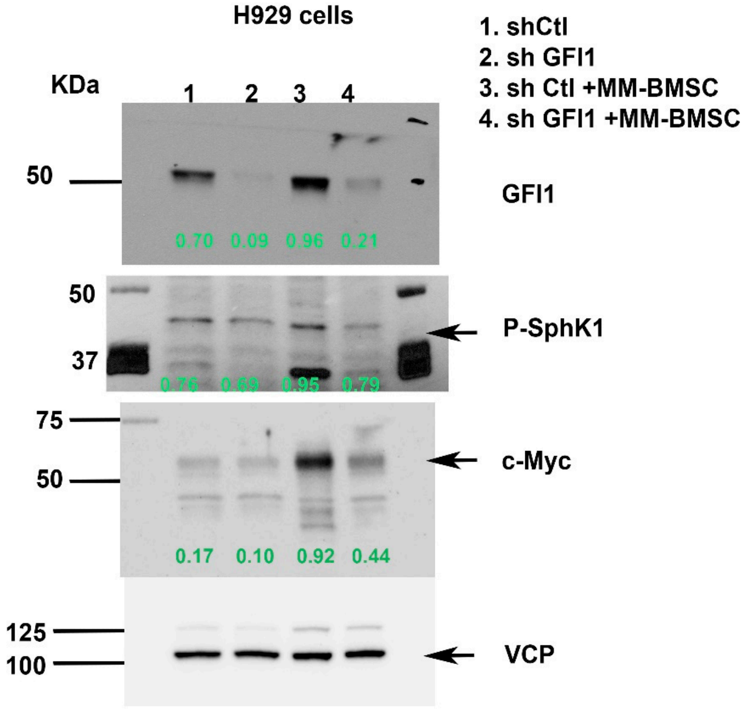

densitometry normalized by VCP loading control

Figure 3 G

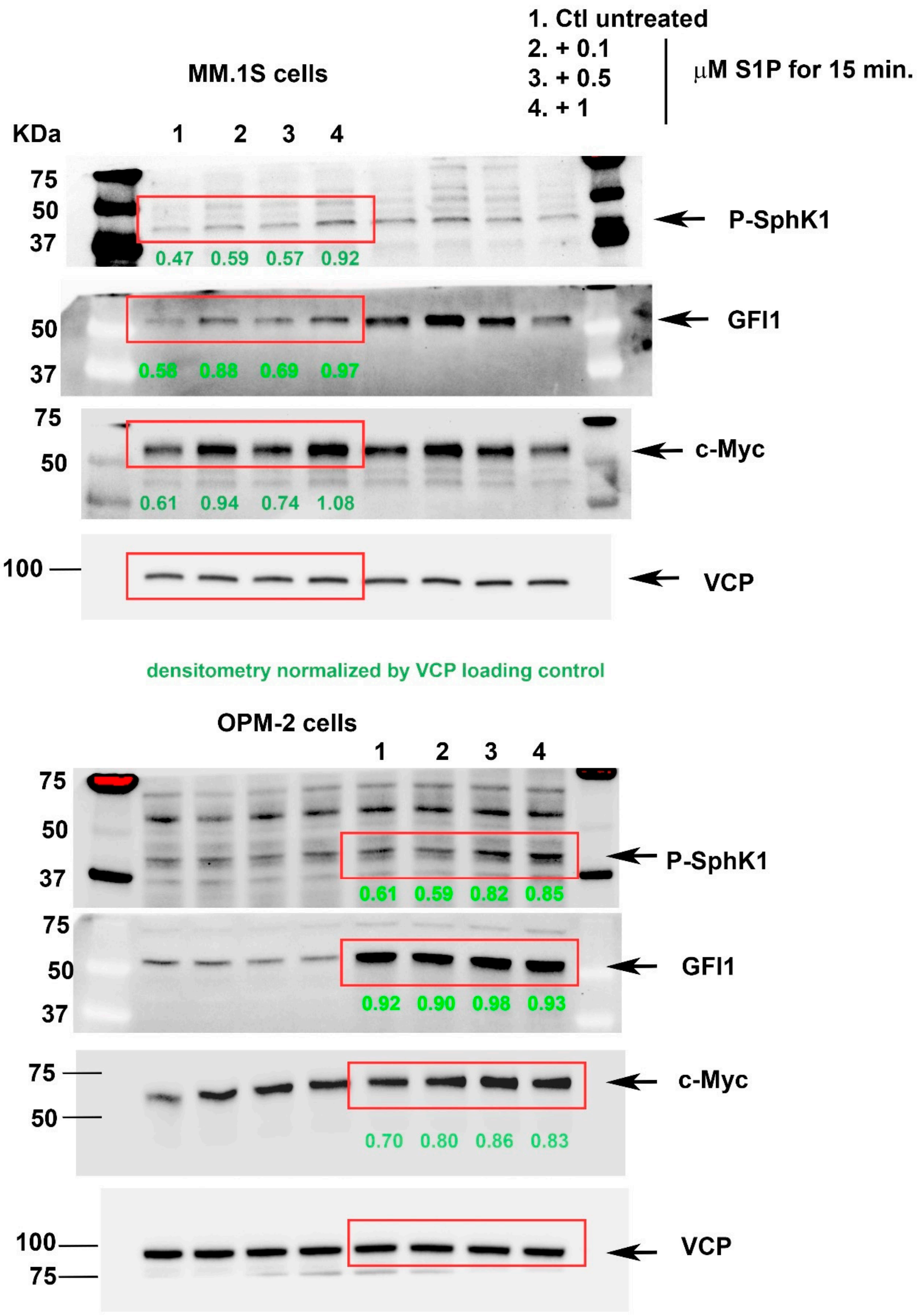
1. Ctl untreated
2. + 0.1
3. + 0.5
4. + 1
μM S1P for 15 min.
MM.1S cells
KDa
1 2 3 4
75
50
37
0.47 0.59 0.57 0.92
P-SphK1
50
37
0.58 0.88 0.69 0.97
GFI1
75
50
0.61 0.94 0.74 1.08
c-Myc
100
VCP
densitometry normalized by VCP loading control
OPM-2 cells
1 2 3 4
75
50
37
0.61 0.59 0.82 0.85
P-SphK1
75
50
37
0.92 0.90 0.98 0.93
GFI1
75
50
0.70 0.80 0.86 0.83
c-Myc
100
75
VCP

Figure 6 A left panel

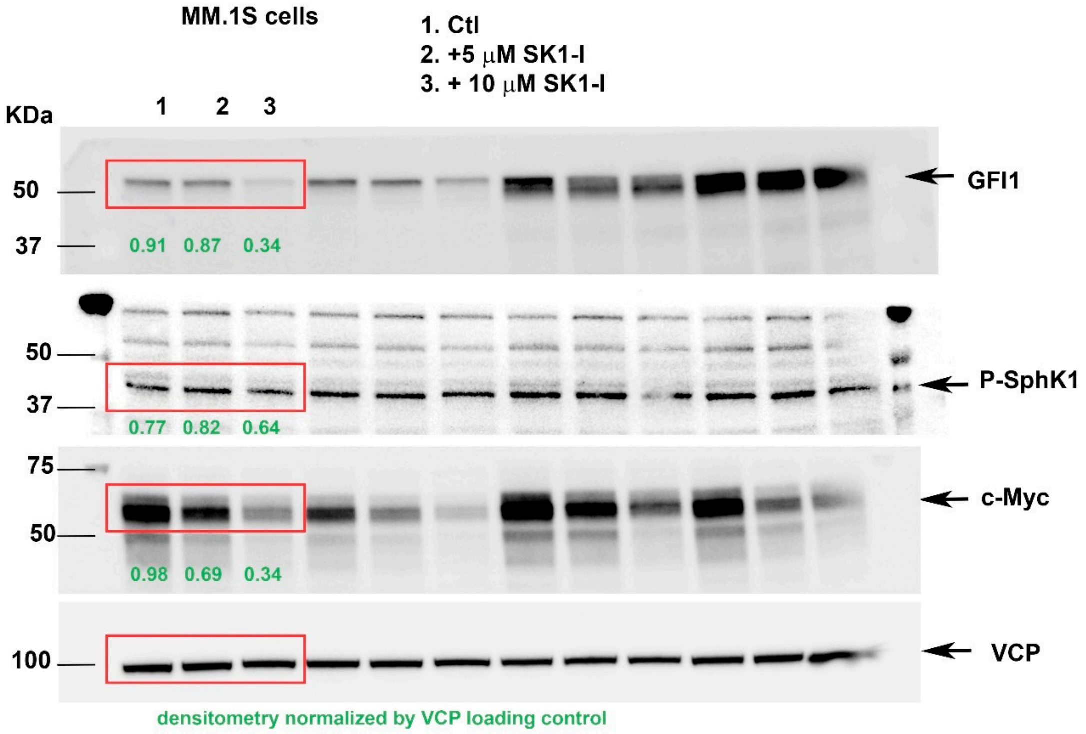

Figure 6 A middle panel

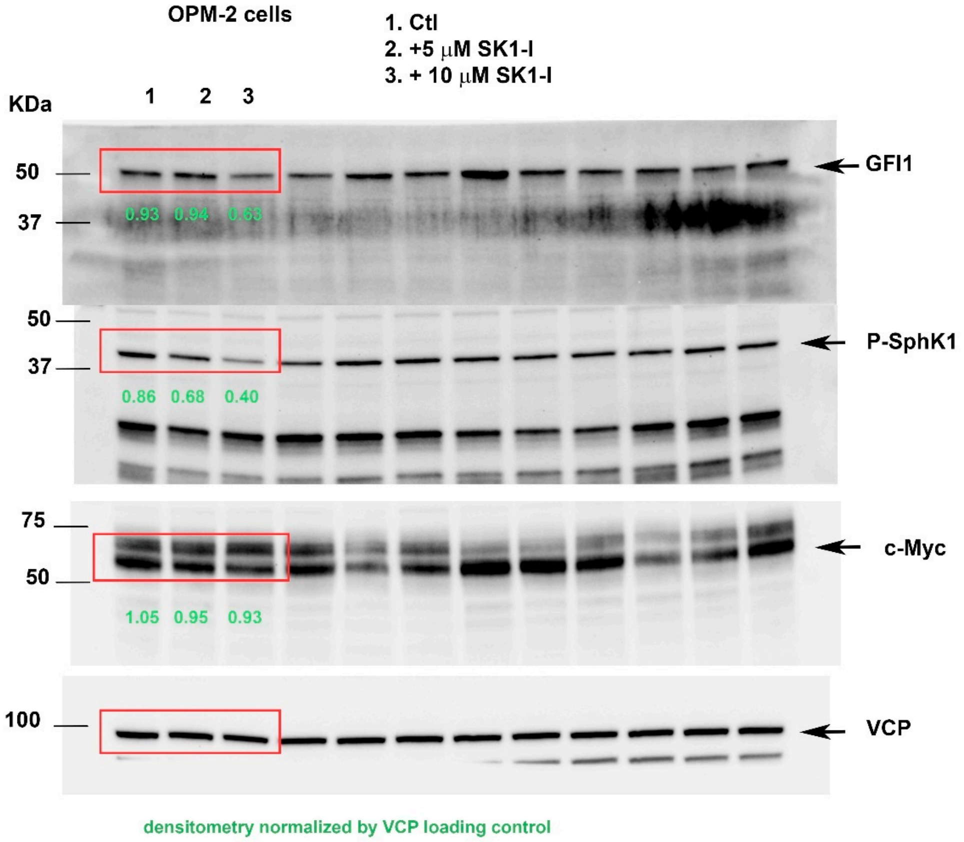

Figure 6 A right panel

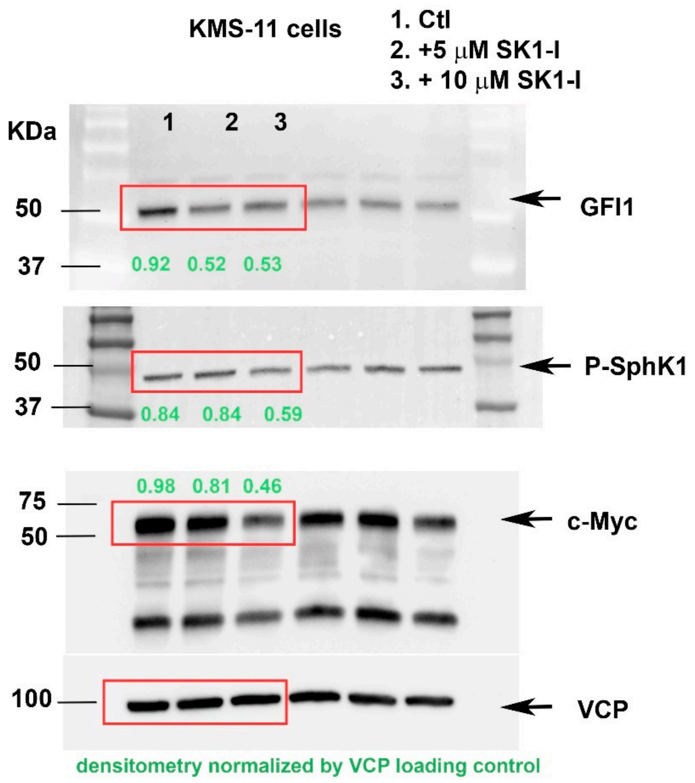

Figure 6 B left panel

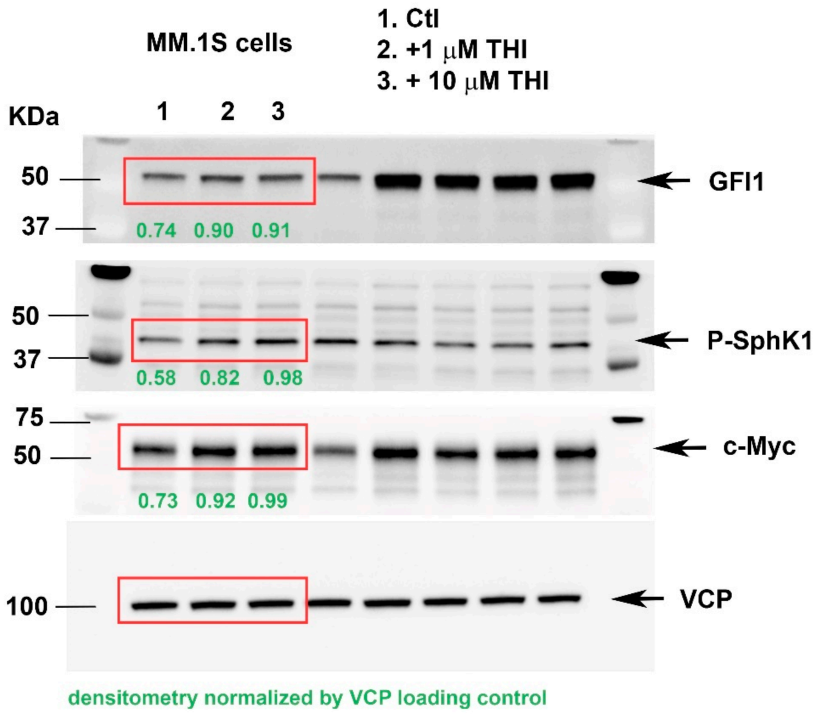

Figure 6 B right panel

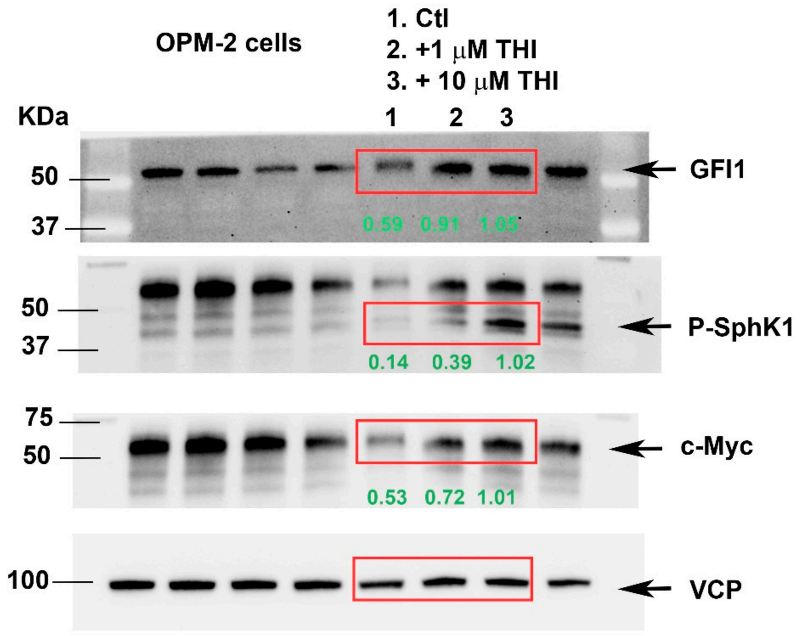

densitometry normalized by VCP loading control

Figure 6 D left panel

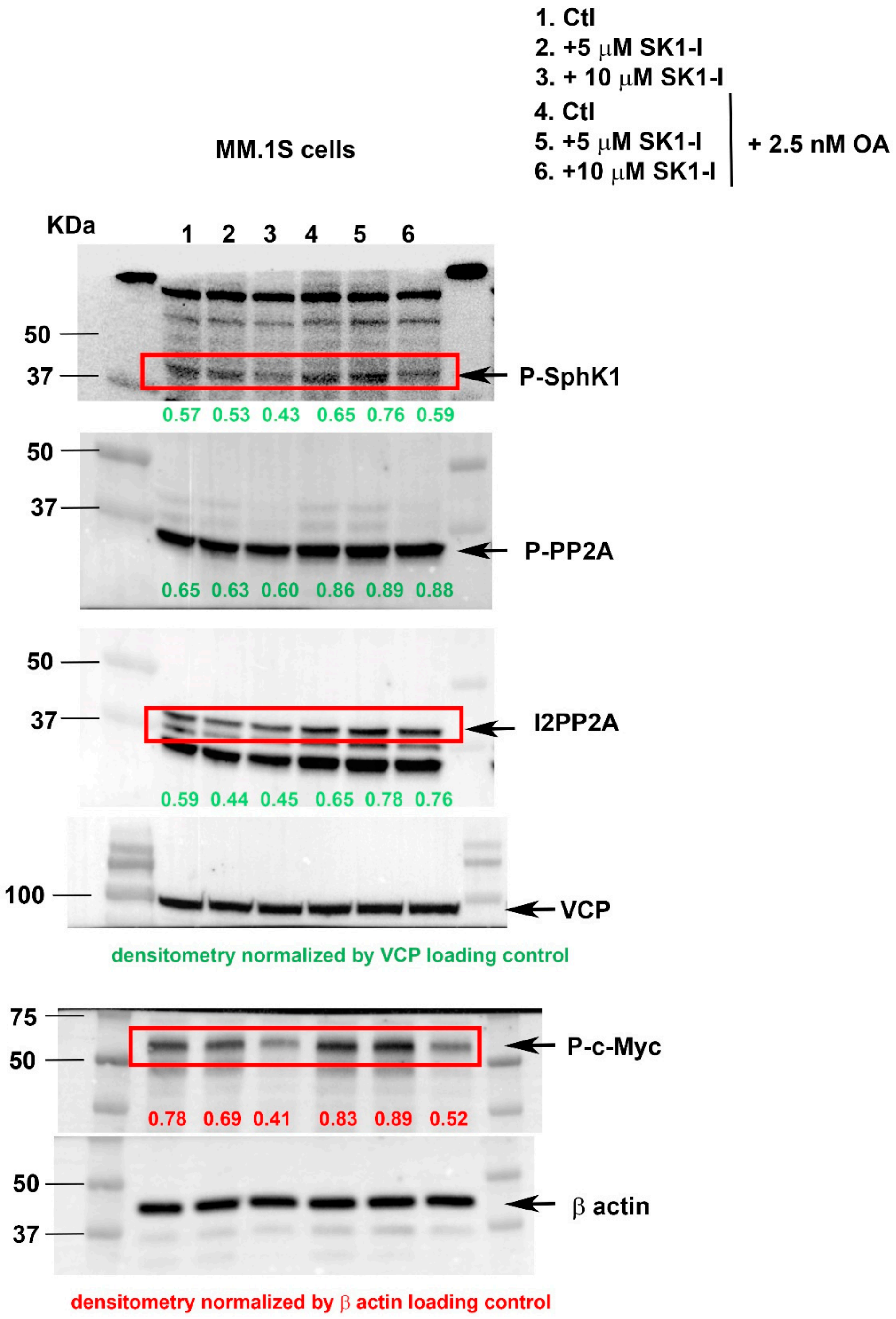

Figure 6 D right panel

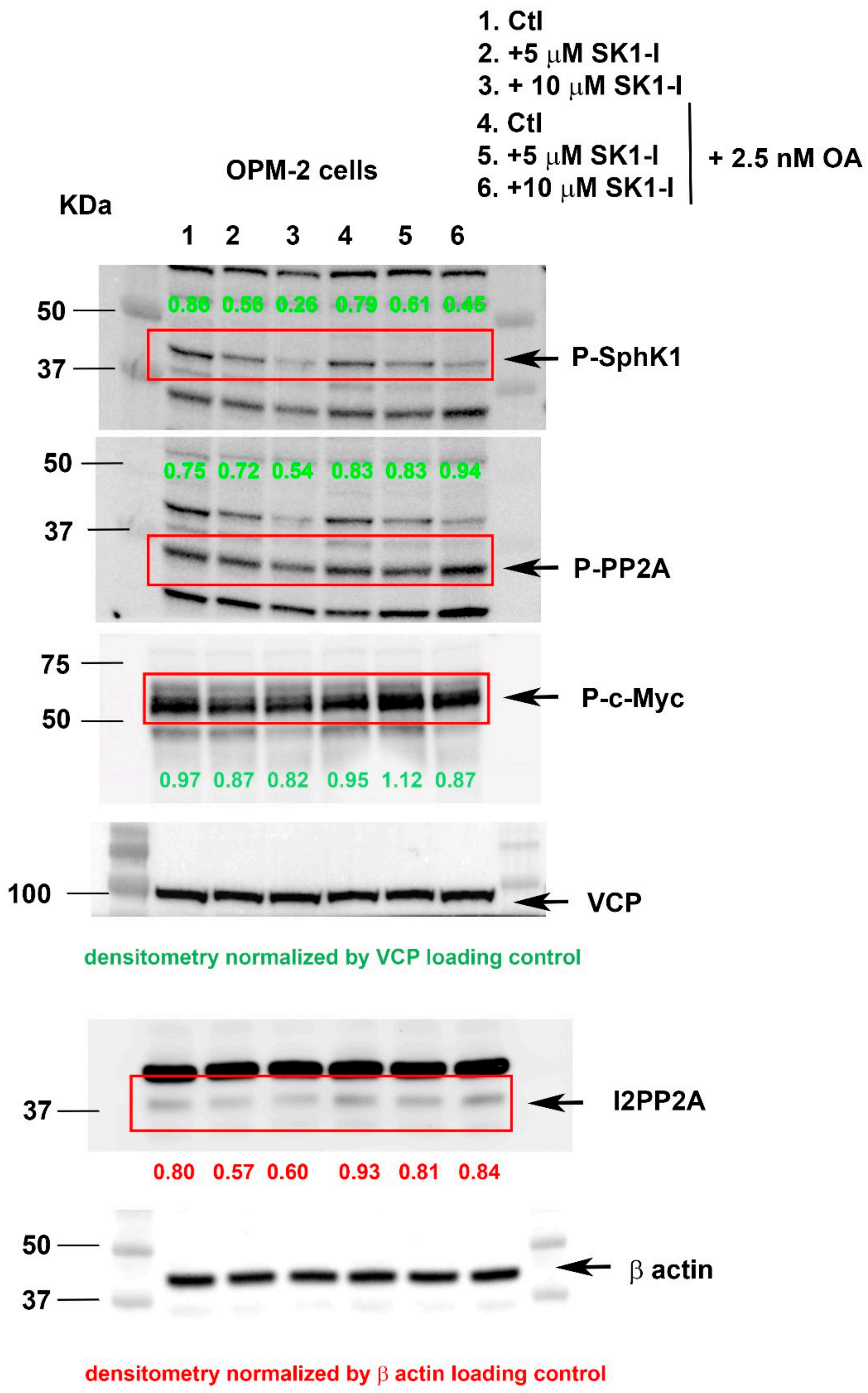

Figure 6 F

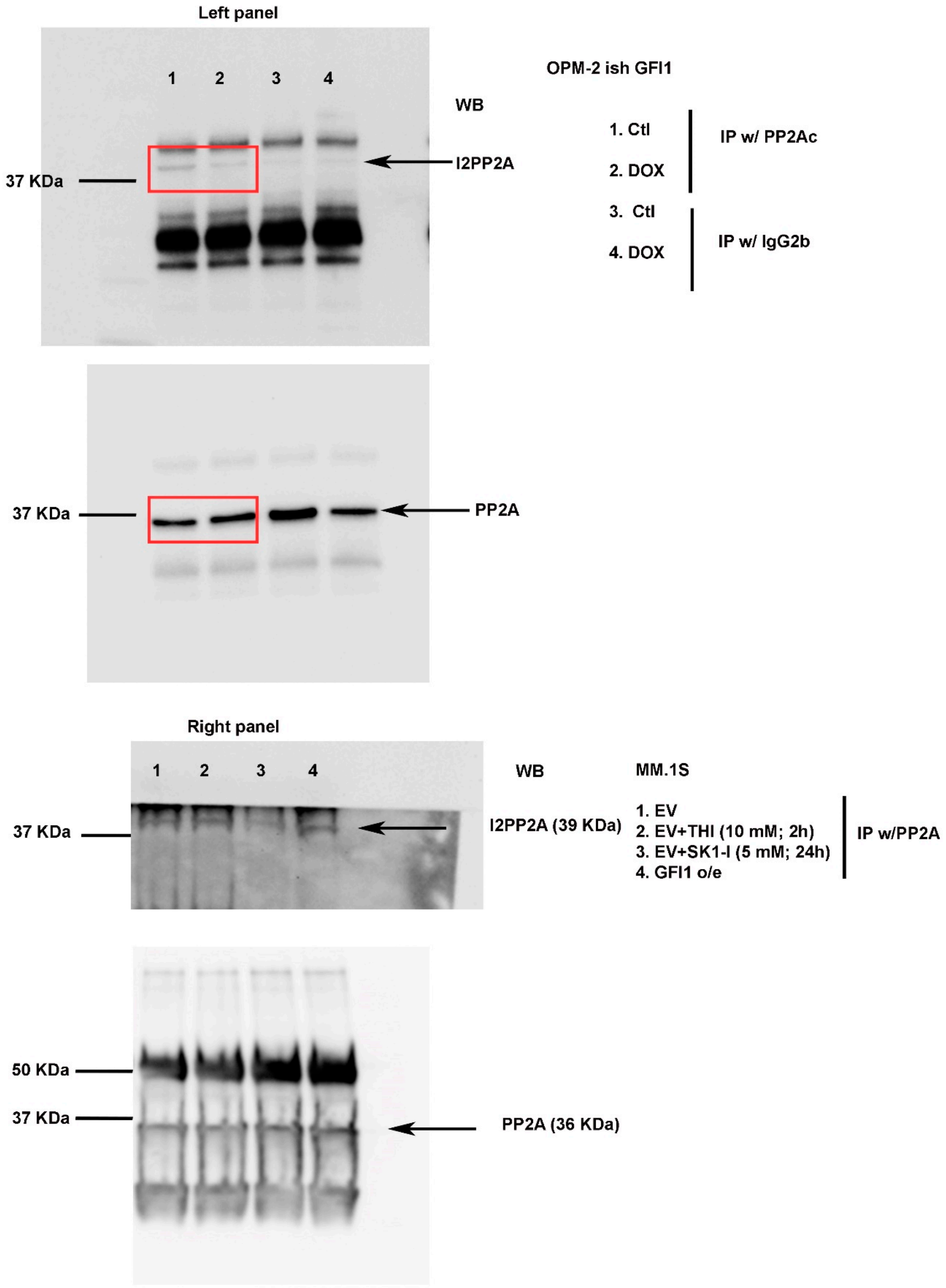
Left panel
1
2
3
4
WB
OPM-2 ish GFI1
1. Ctl
2. DOX
IP w/ PP2Ac
3. Ctl
4. DOX
IP w/ IgG2b
37 KDa
I2PP2A
37 KDa
PP2A
Right panel
1
2
3
4
WB
MM.1S
37 KDa
I2PP2A (39 KDa)
1. EV
2. EV+THI (10 mM; 2h)
3. EV+SK1-I (5 mM; 24h)
4. GFI1 o/e
IP w/PP2A
50 KDa
37 KDa
PP2A (36 KDa)

Figure 6 H left panel

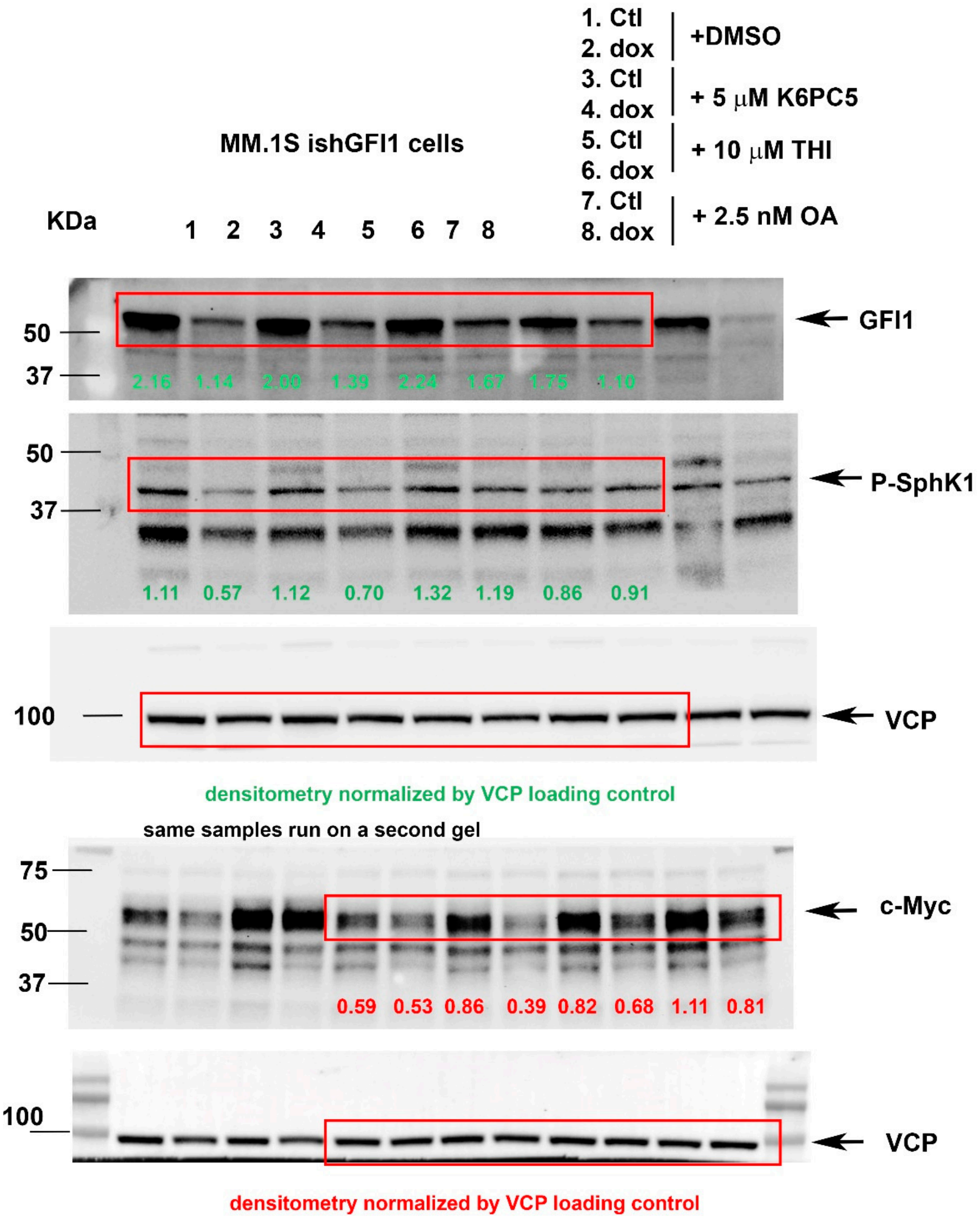

Figure 6 H right panel

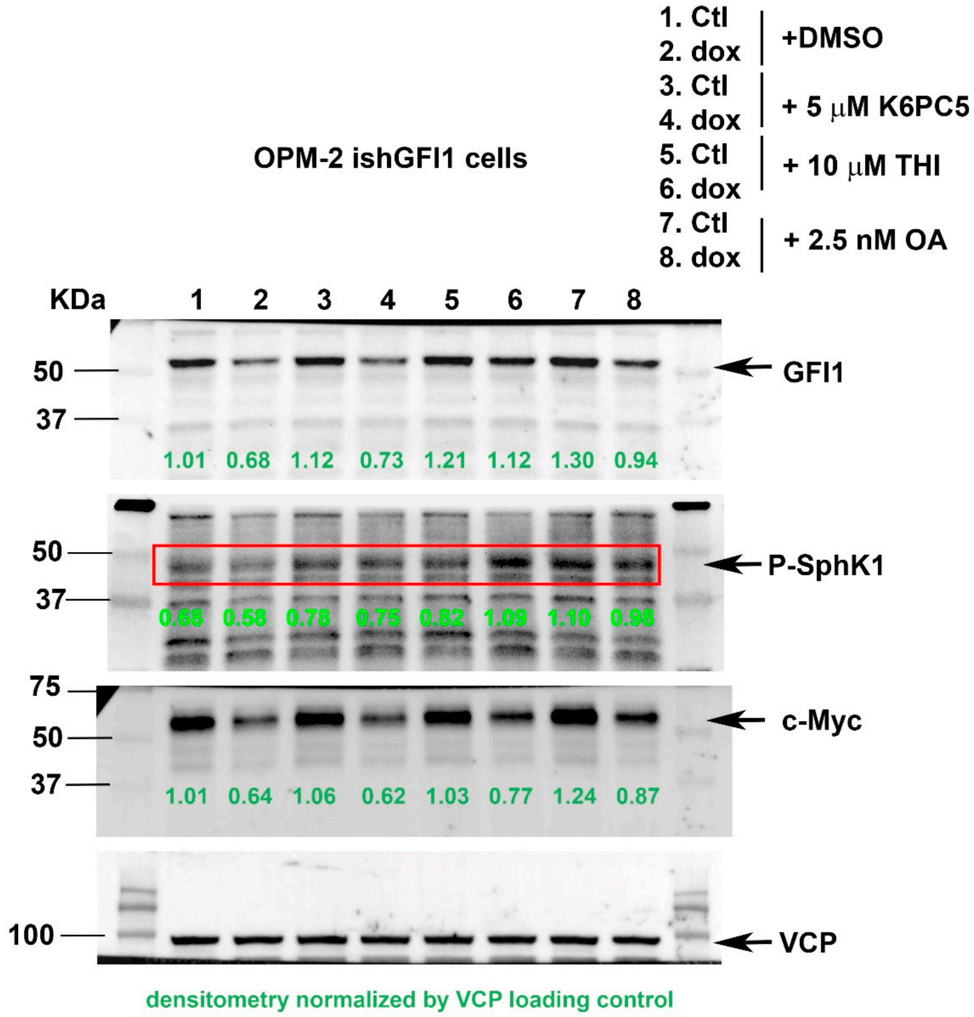

Figure 7 A left panel

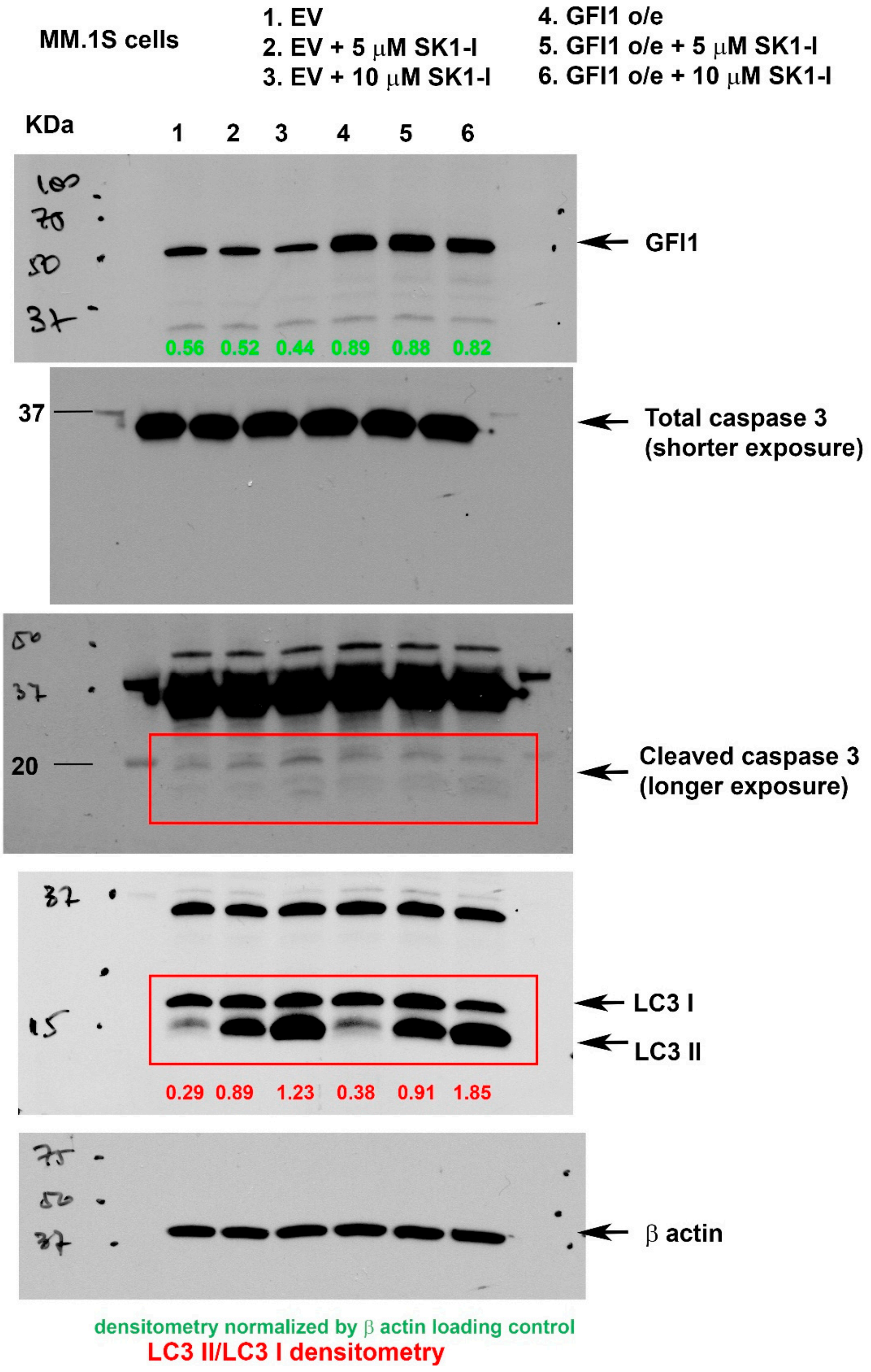

Figure 7 A right panel

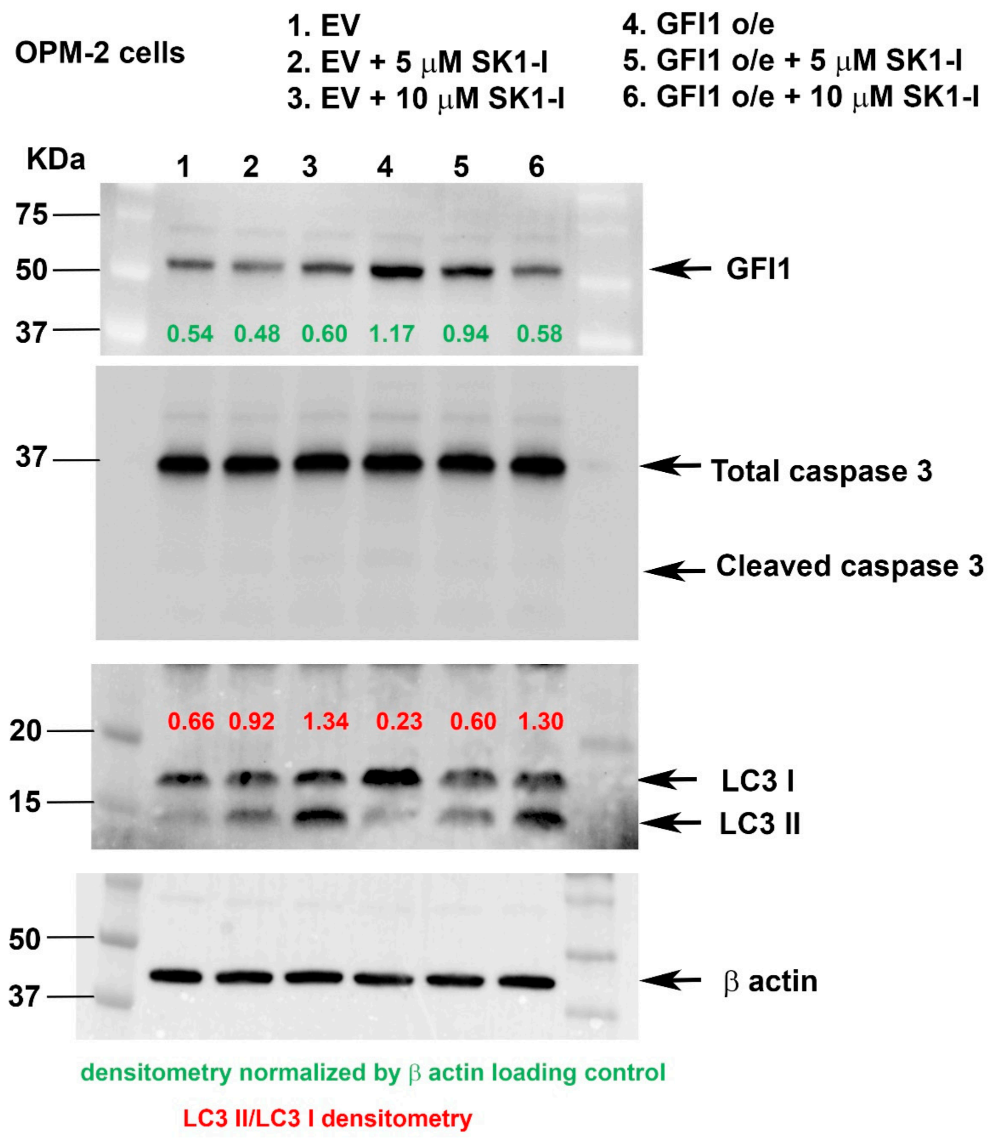

Figure 7 B left panel

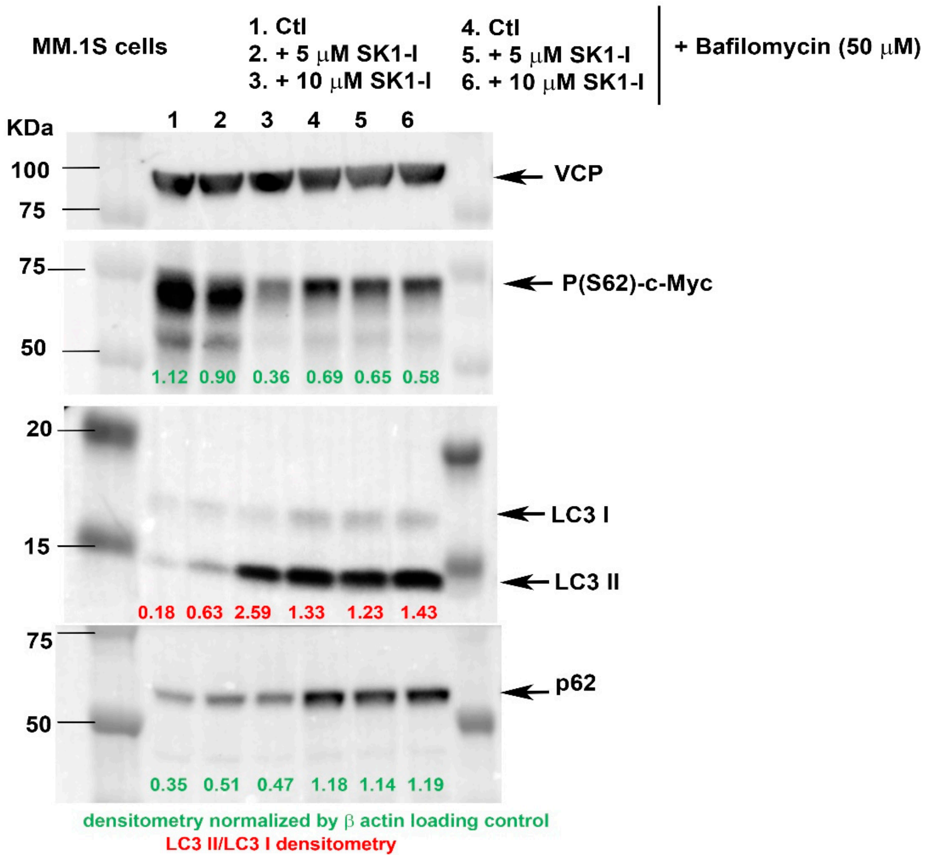

Figure 7 B right panel

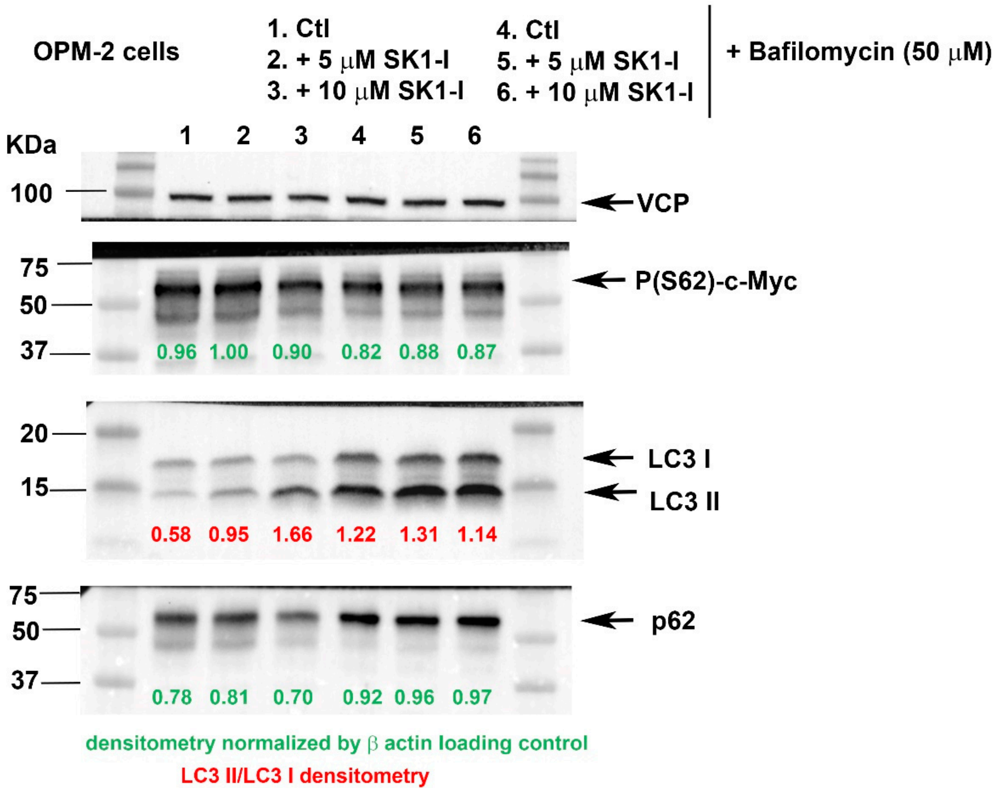

Figure S4 D left and middle panels

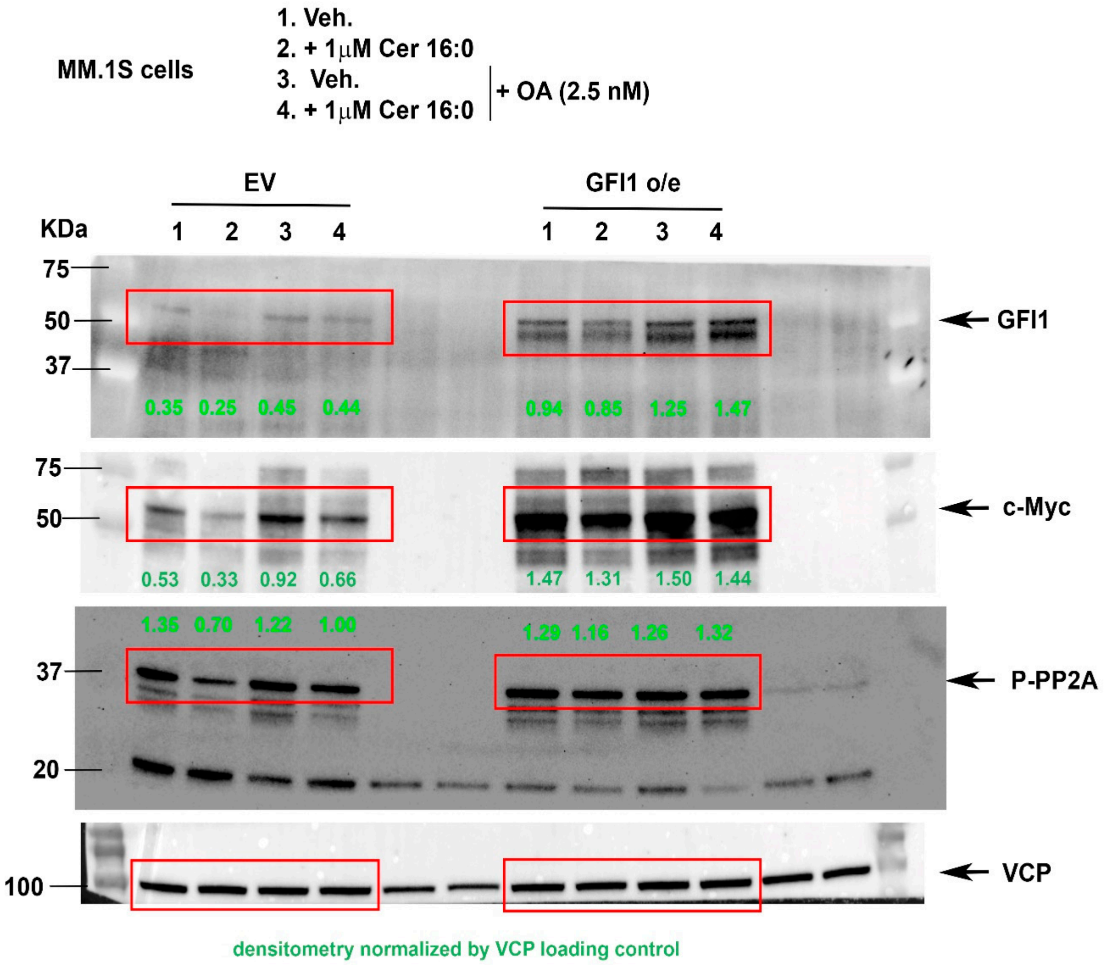

Figure S4 D right panel

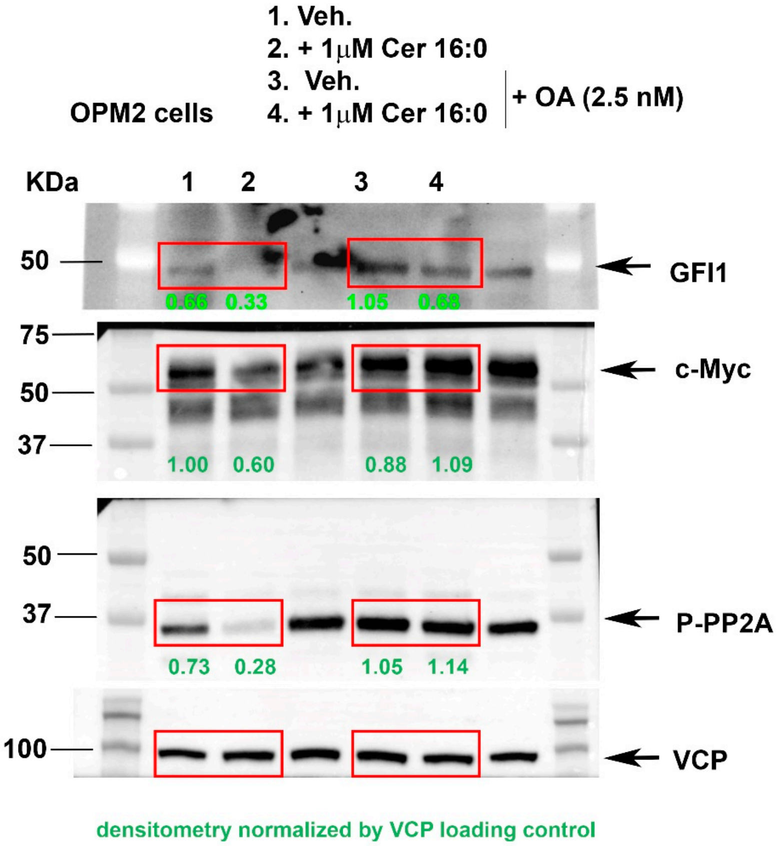

Figure S4 E left and right panel

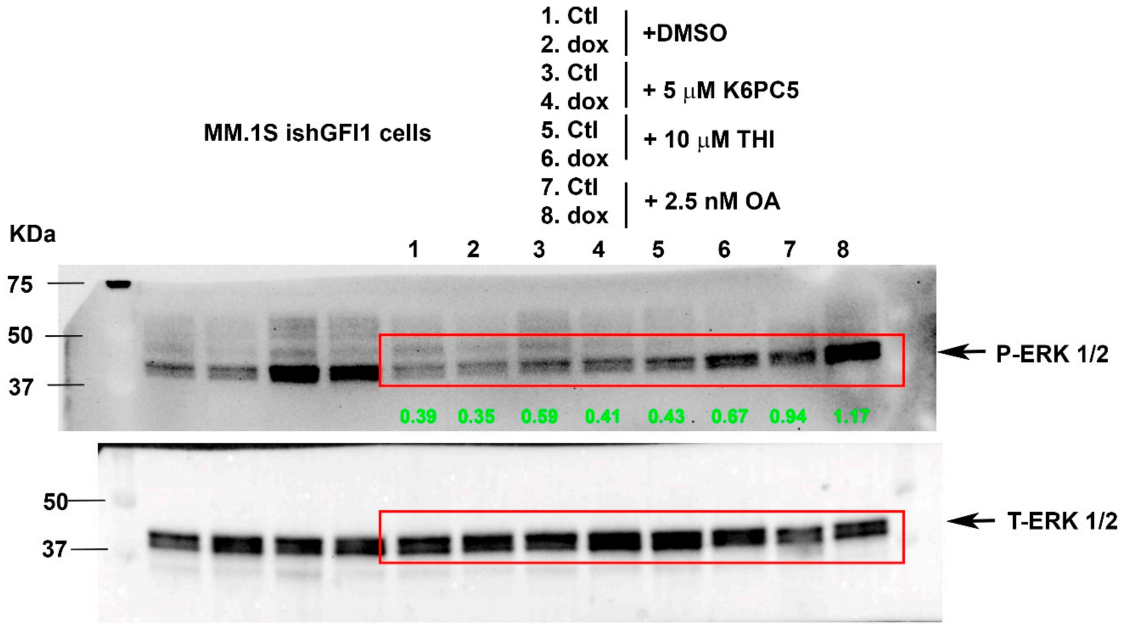

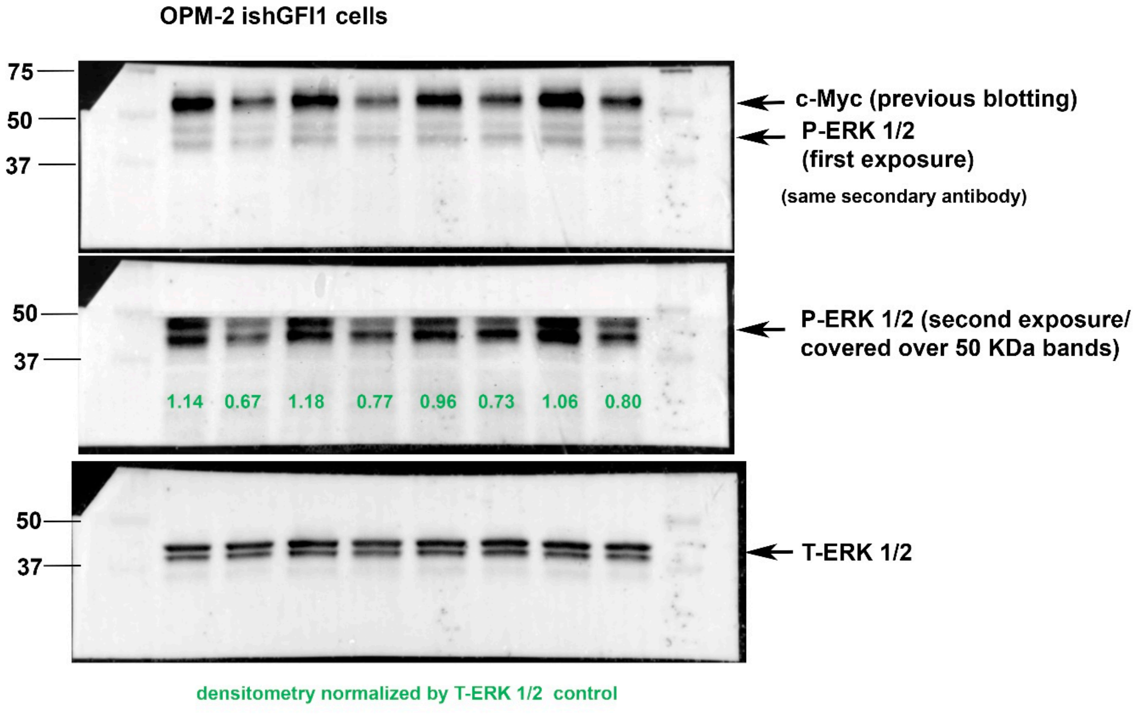

Figure S4 F left panel

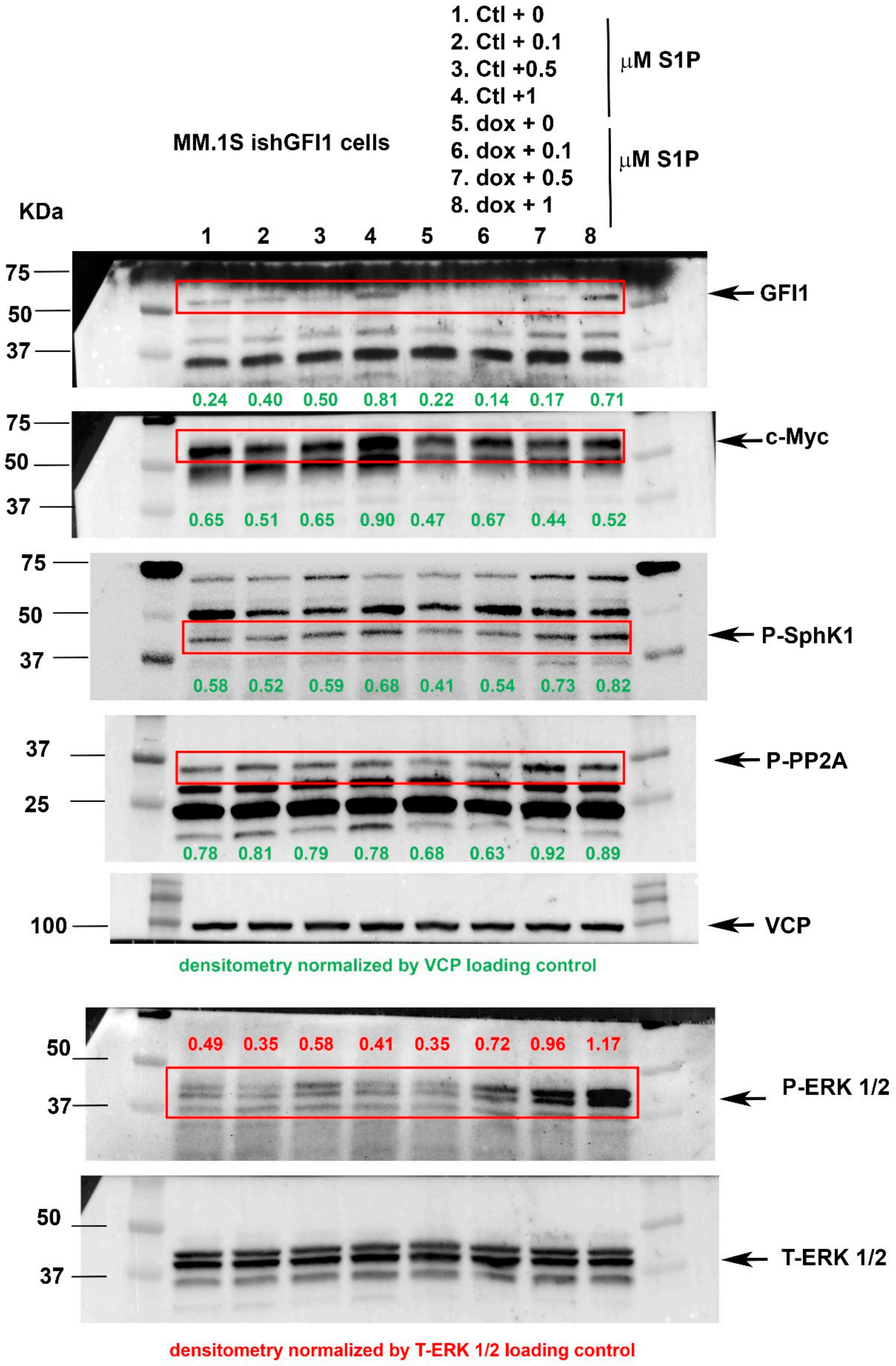

Figure S4F right panel

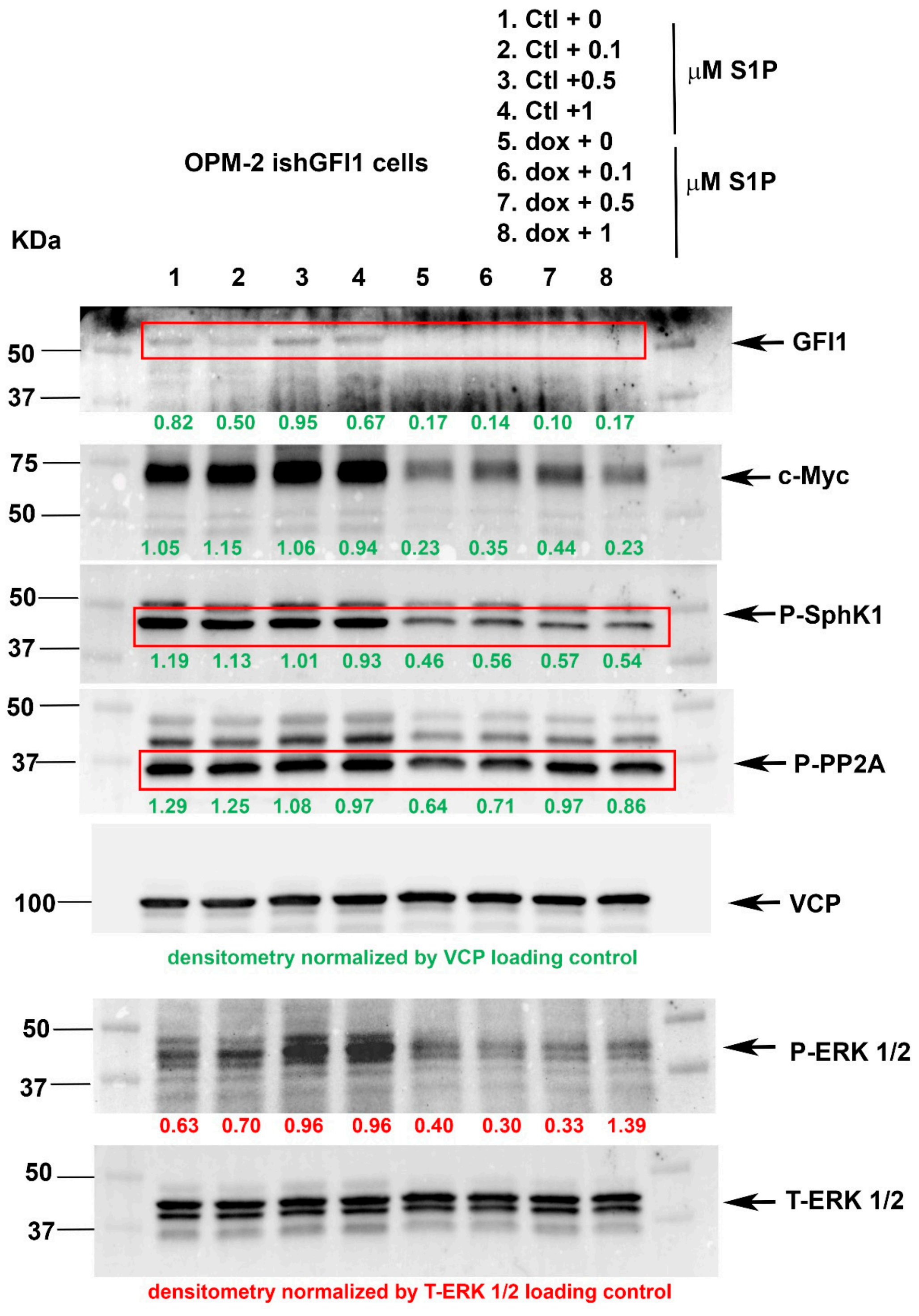

Supplement: Supplementary file 1 [file cancers-14-00772-s001.zip › Supplementary Figure S6_Petrusca et al_Cancers 2022.pdf]
