# Supplementary material for: GFI1-Dependent Repression of SGPP1 Increases Multiple Myeloma Cell Survival
Source: Cancers (Basel). 2022 Feb 2;14(3):772. doi: 10.3390/cancers14030772 (PMC8833953; doi:10.3390/cancers14030772)
Supplement: Supplementary file 1 [file cancers-14-00772-s001.zip › Supplementary Figures 1-5_Petrusca et al_Cancers 2022-better resolution.pdf]

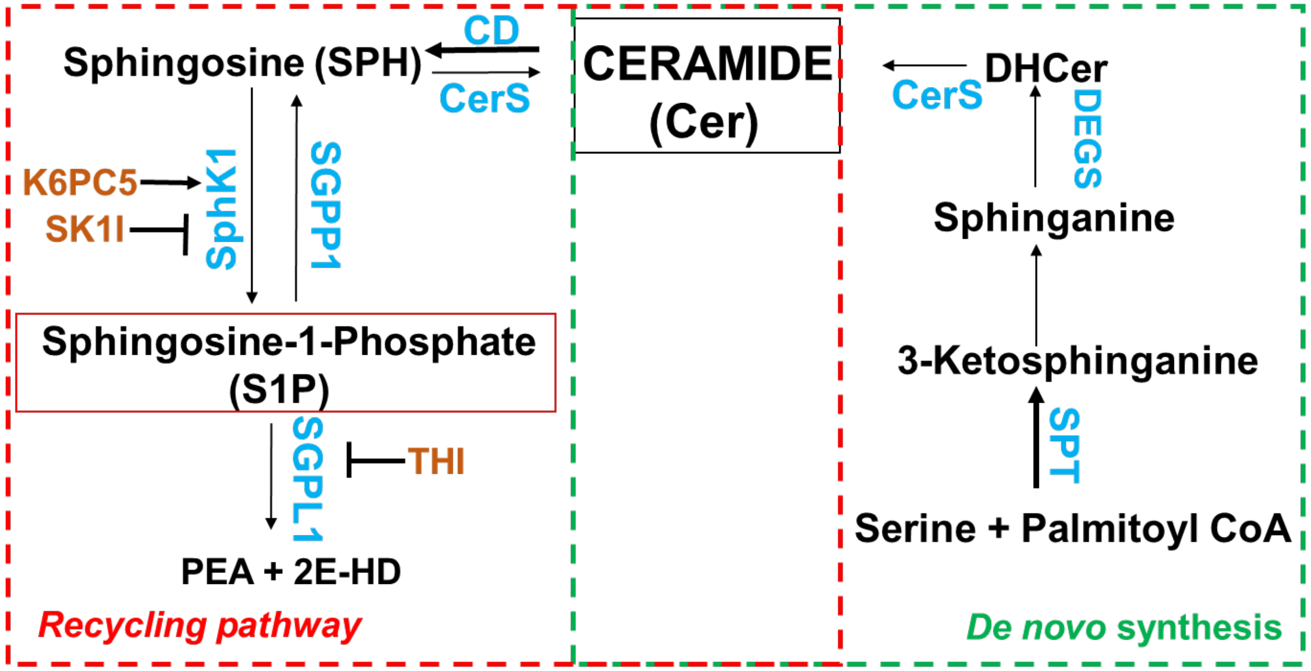

Figure S1. Schematic of biochemical pathways of sphingolipid regulation (enzymes that regulate each step and their specific inhibitors or activators).

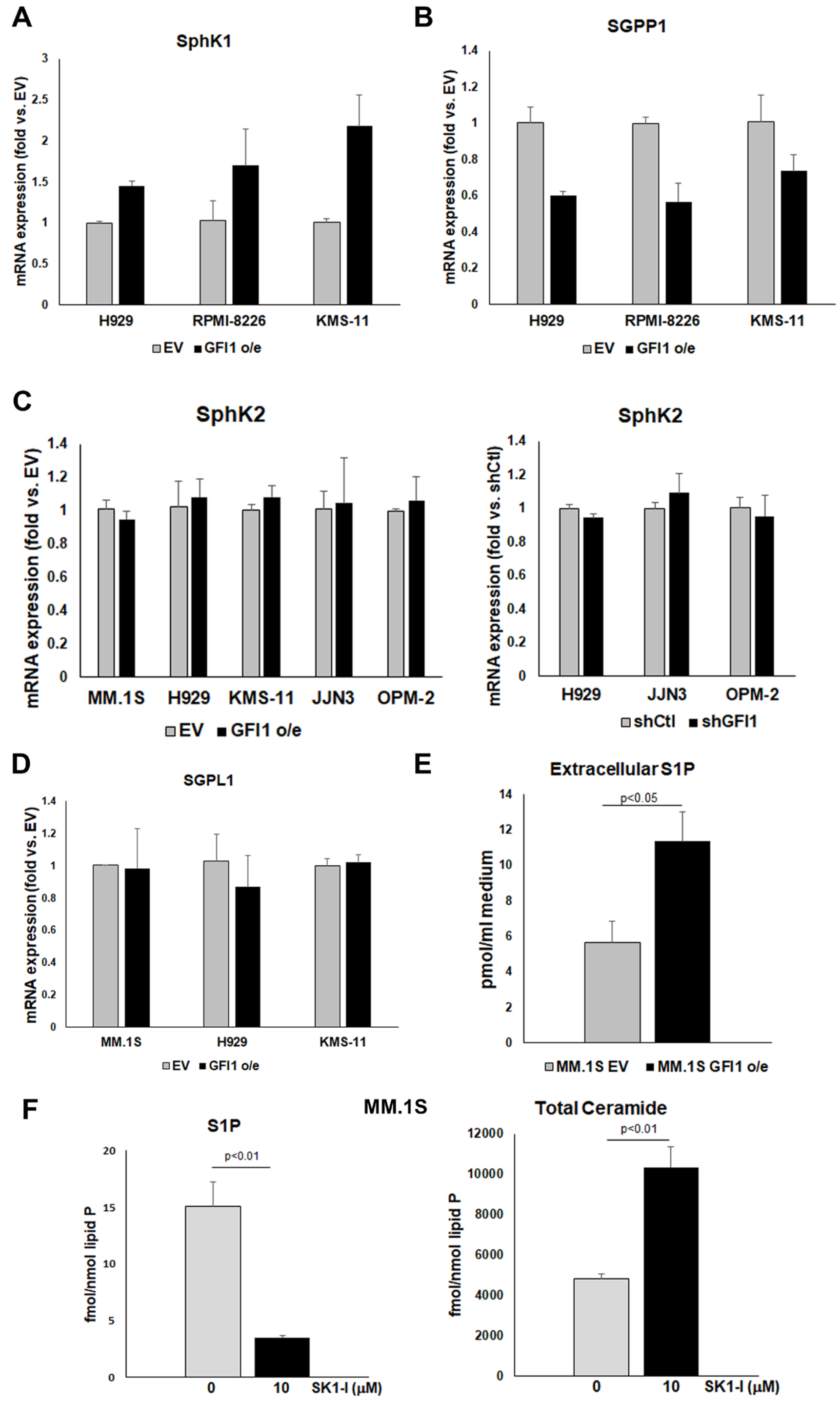

Figure S2. SphK2 and SGPL1 are not modulated by GFI1 in MM cells. The mRNA levels of different genes were measured by qPCR and expressed as fold change versus respective control cells as follows: *SphK1* (A) and *SGPP1* (B) in stable GFI1 overexpressing (Go/e) H929 (p53 WT), RPMI-8226 (p53 mut) and KMS-11 (p53 null) cells; *SphK2* in stable G o/e cells (MM.1S, H929, KMS-11, JJN3 and OPM-2) (C-left panel) and in stable ishGFI1 expressing cells (H929, JJN3 and OPM-2) treated for 3 days with 1 μg/ml dox (C-right panel); *SGPL1* in stable Go/e cells (MM.1S, H929 and KMS-11) (D); Abundance of sphingolipid species as measured by LC-MS/MS in MM.1S cells as follows: S1P levels in the supernatant of stable GFI1 o/e cells compared to their EV controls (E) and S1P (F-left panel) and total ceramide (F-right panel) after 24h treatment with SK1-I (10 μM). Data are the average of three biological replicates (Mean +/-SEM) ($p<0.05$ vs. either EV control (E) or untreated control (F).

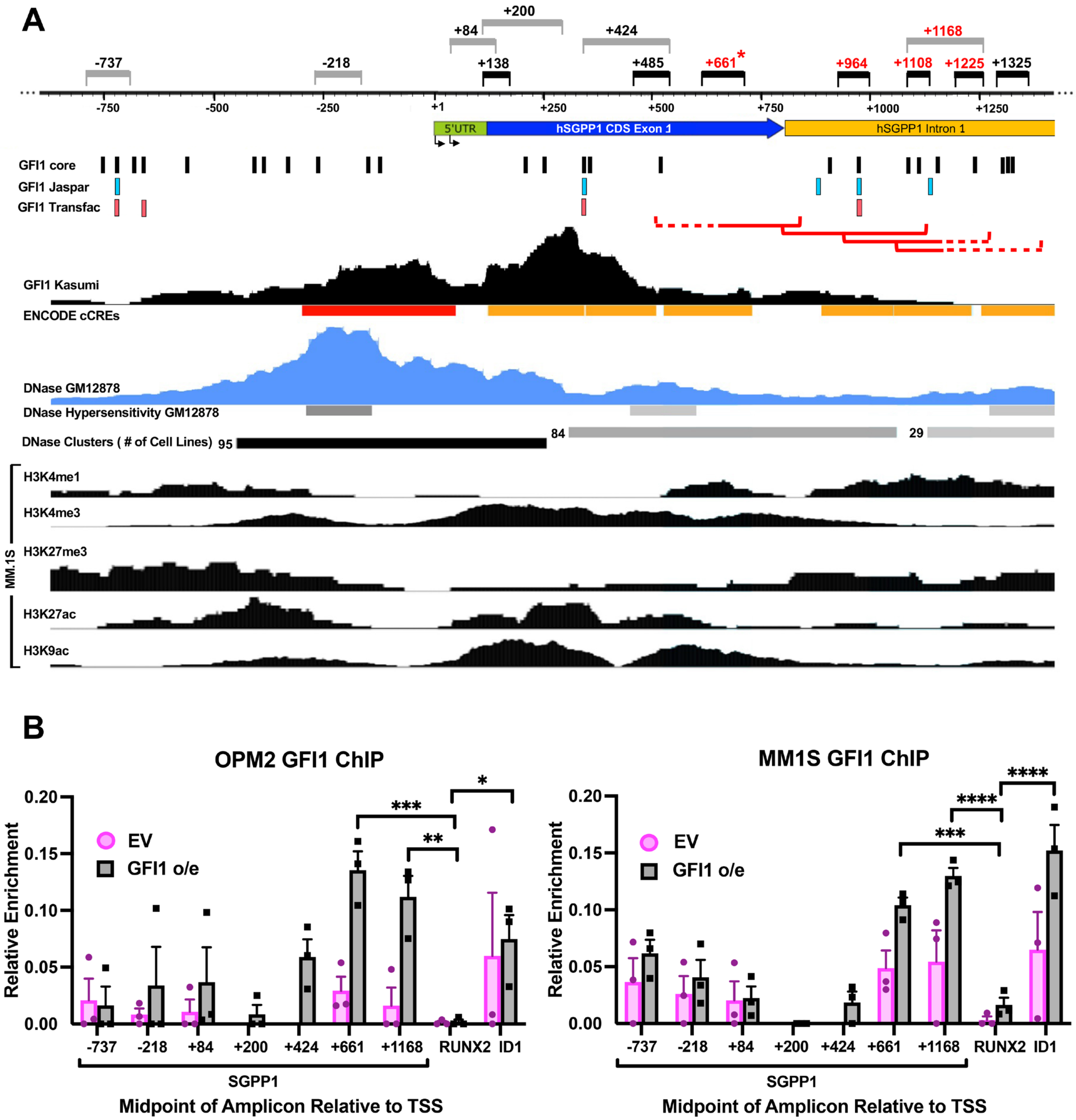

Figure S3. Preliminary ChIP-qPCR scan using *SGPP1* 500-bp chromatin fragments from MM cells reveals GFI1 binding. A schematic of the promoter, exon1 and part of intron1 of human *SGPP1* NM_030791.4 (GRCh38.p12; GCF_000001405.27) is presented below a nucleotide scale labeled with respect to the *SGPP1* TSS (+1) at nucleotide 63,728,065. PCR amplicons (midpoints relative to *SGPP1* TSS) used to analyze GFI1 ChIP-qPCR using 500-bp (Gray bars) or 200-bp (Black bars) chromatin fragmentation; red amplicon # has significant results in this study. *, +661 amplicon was used in both screens. The predicted putative GFI1 binding sites found using searches for the **GFI1 core** AA(T/G)C; or the **Jaspar**; and **Transfac** algorithms are shown, along with red brackets that indicate the detection ranges of the positive amplicons (dashes; overlaps with the detection range of a negative amplicon). CistromeDB BigWig files loaded into UCSC Genome browser tracks [53, 54] are shown for the h*SGPP1*. Tracks:

GFI1 Kasumi, GFI1 ChIP-seq using the Kasumi-1 AML cell line [91] (#90189); **Encode cCREs**, UCSC track for ENCODE cCREs: red bars marking likely promoters and orange bars likely enhancers [55, 57]; **DNase GM12878** B-cell lymphoblastoid cell-line (blue peaks) and **DNase GM12878 Hypersensitivity** (gray bars), USCS DNase I hypersensitive-seq scan analyses [58, 59]; The **DNase Clusters** track shows the DNase I Hypersensitivity Peak Clusters from all 95 ENCODE cell types [60, 61]; **MM.1S** tracks showing histone modifications: **H3K4me1** (#62686), **H3K4me3** (#33169), **H3K27me3** (#43093), **H3K27ac** (#33183), and **H3K9ac** (#64725) (A);. Chromatin from OPM2 and MM1S cells stably overexpressing GFI1 or EV control was fragmented to 500-bp. qPCR was performed and binding was compared to a background control amplicon on the *RUNX2* gene (+66065) and a positive control amplicon on the *ID1* gene (+275). Statistical analyses used one-way ANOVA with Dunnett's correction for multiple comparisons (*$p < 0.05$; **$p < 0.01$; ***$p < 0.001$; ****$p < 0.0001$) (B).

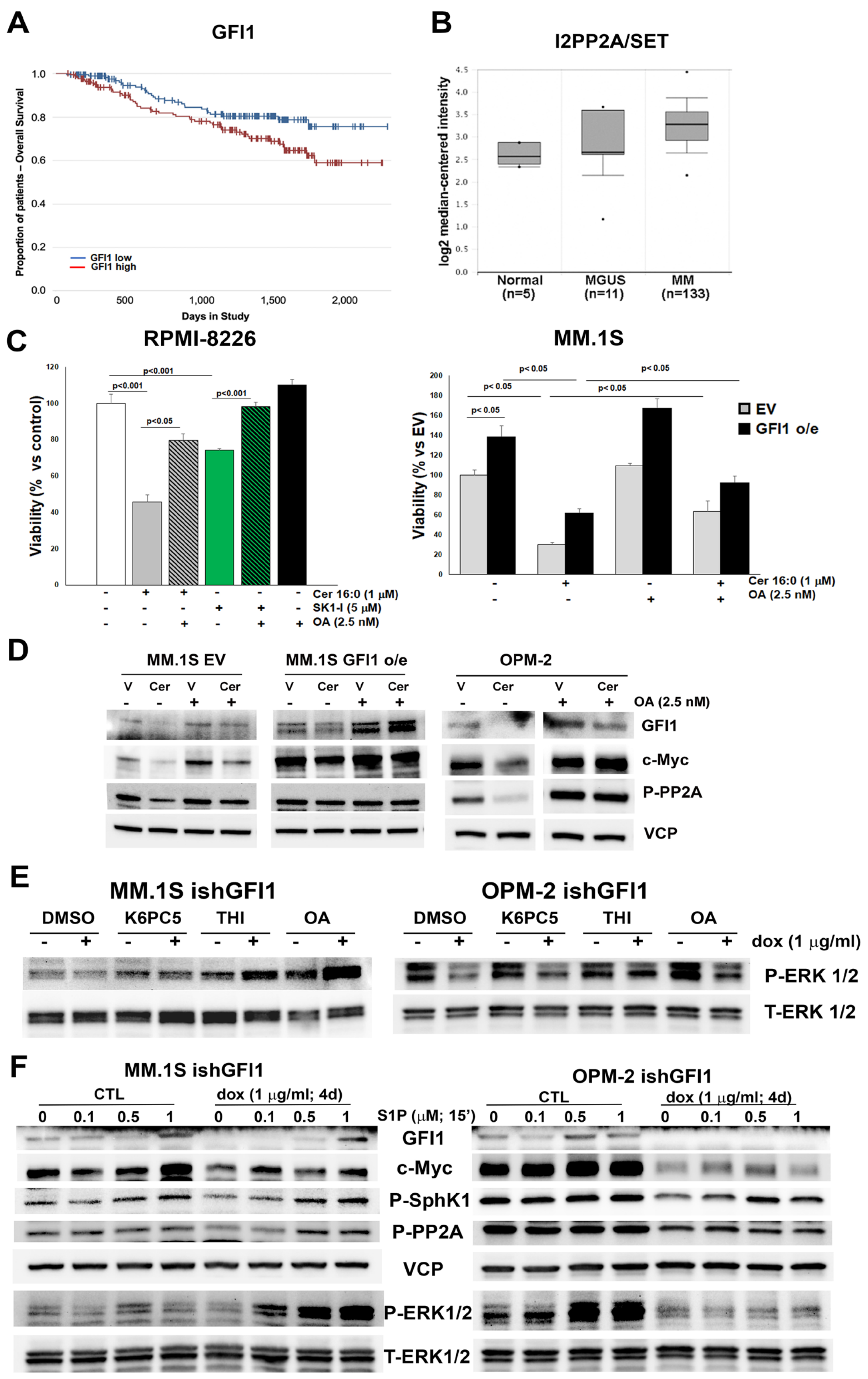

Figure S4. S1P activates ERK - SphK1 axis to increase c-Myc stability in a PP2A dependent manner. Overall survival based on *GFI1* mRNA expression (lower quartile expression in blue, n=193 and upper quartile in red, n= 193) of newly diagnosed MM patients using the MMRF IA15 dataset (p=0.0015 by Log-rank test) (A); Samples from CD138+ purified normal (n=5), MGUS (n=11) and multiple myeloma (n=133) corresponding to Agnelli Myeloma 3 dataset (41) were analyzed for *I2PP2A/SET* (p=9.53E-29 normal vs. MM and p=0.005 normal vs. MGUS) (B); RPMI-8226 (p53 mut) (left panel) and MM.1S (p53WT) EV and GFI1 o/e (right panel) cells, were treated with either

SK1-I (5 μM), Cer16:0 (1 μM) or vehicle control (DMSO) after being pre-treated or not with OA (2.5nM, 2h). Viability was measured by AlamarBlue assay after 48h and reported as percent vs. vehicle treated control (C); MM.1S EV and GFI1 o/e (left and middle panels) and OPM-2 (right panel) cells were treated with either Cer16:0 (1 μM) or vehicle control (DMSO) for 24h after being pre-treated or not with OA (2.5nM, 2h). Whole cell lysates were probed by WB with specific antibodies and presented by representative immunoblots (D); KD GFI1 was induced in MM.1S ishGFI1 (left panels) and OPM-2 ishGFI1 (right panels) cells by dox treatment (1 μg/ml) for 3 days after which were treated with THI (10 μM), K6PC5 (5 μM), OA (2.5 nM) or vehicle (DMSO) for an additional 24h (E) or with S1P (0.1, 0.5 or 1 ◎M) or vehicle (ethanol) for 15 min (F). Whole cell lysates were probed by WB with specific antibodies and presented by representative immunoblots (E and F).

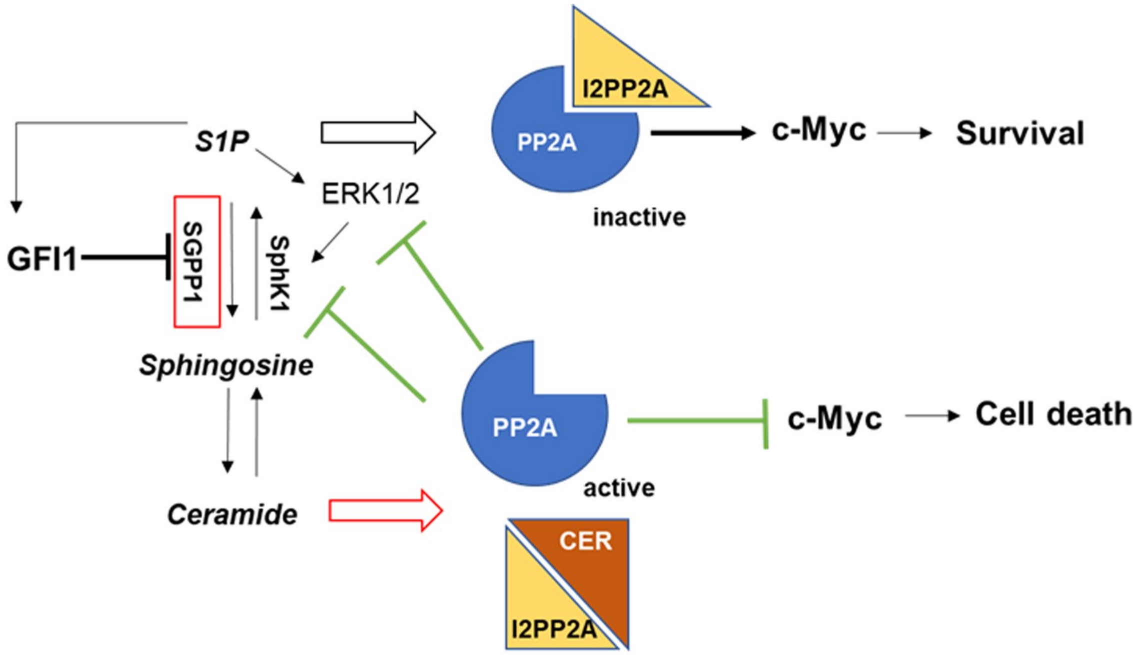

Figure S5. Schematic of the proposed mechanism. GFI1 represses *SGPP1* expression, thus maintaining high intracellular S1P levels that keep PP2A inactive leading to high c-Myc protein levels and MM cell survival. High intracellular S1P levels also activate a ERK1/2-dependent positive feedback loop that prevents PP2A activation. When the Cer:S1P ratio increases, ceramide binds I2PP2A and, thus active PP2A can inhibit c-Myc as well as ERK ½ -dependent activation of SphK1 leading to MM cell death.
