# Supplementary material for: GFI1-Dependent Repression of SGPP1 Increases Multiple Myeloma Cell Survival
Source: Cancers (Basel). 2022 Feb 2;14(3):772. doi: 10.3390/cancers14030772 (PMC8833953; doi:10.3390/cancers14030772)
Supplement: Supplementary file 1 [file cancers-14-00772-s001.zip › Supplementary Tables _Petrusca et al_Cancers 2022.pdf]

Table S1: Main characteristics of the MM Patients cohort

Patient	*Clinical features*					
ID	*Age (years)*	*Gender*	*Race*	*Newly diagnosed*	*ISS Stage*	*Skeletal disease*
MM 1	55	M	white	N	III	Y
MM 2	60	F	white	N	III	Y
MM 3	73	M	white	N	I	N
MM 4	55	M	white	N	III	Y
MM 5	72	F	white	N	I	Y
MM 6	76	M	white	N	I	Y
MM 7	62	F	white	N	I	Y
MM 8	67	F	white	N	I	Y
MM 9	54	F	white	N	I	Y
MM 10	60	M	white	Y	I	N

Table S2: Main characteristics of the Normal donor cohort

Normal Donor ID	*Age (years)*	*Gender*	*Race*
ND 1	33	F	white
ND 2	21	F	white
ND 3	23	M	white

Abbreviations: MM- Multiple Myeloma; ND- normal donor.

Table S3: Sequences of qPCR primers used for amplification of human mRNA

qPCR	Gene	Primer	Sequence 5’-3’
	GFI1	Forward	GAGCCTGGAGCAGCACAAAG
		Reverse	GTGGATGACCTCTTGAAGCTCTTC
	SphK1	Forward	TCTGGTGGTCATGTCTGGAG
		Reverse	CACAGCAATAGCGTGCAGTT
	SphK2	Forward	GGTTGCTTCTATTGGTCAATCC
		Reverse	GTTCTGTCGTTCTGTCTGGATG
	SGPP1	Forward	CGCTGGCAGTACCCTCTTAT
		Reverse	GAAGCCCGATGATGATGAAT
	c-Myc	Forward	TTCGGGTAGTGGAAAACCAG
		Reverse	CAGCAGCTCGAATTTCTTCC
	18s	Forward	ATC CCT GAA AAG TTC CAG CA
		Reverse	CCC TCT TGG TGA GGT CAA TG

Table S4: Sequences of primers used in ChIP-qPCR.

Gene (Human)	Amplicon Midpoint Relative to the TSS	Forward Primer 5' to 3'	Reverse Primer 5' to 3'	Amplicon Size (bp)	Detection ranges* on 200-bp fragments
SGPP1	-737	TTGGTAACTTGTG AGACACGGAA	AGAGGGCTTGC ACAGAATAAATG	106	
SGPP1	-218	AGGGAATCCGGC GACACTA	CCGGGTAATAT CCACCAGCAG	112	
SGPP1	+84	TGTGCCGGTTCC GCTG	GCTGCCTCAGC GACATGATA	106	
SGPP1	+138	TTCCGTTATCATG TCGCTGAGG	TGCAGACGGCC AACCAG	54	-35 to +312
SGPP1	+200	TTCCGTTATCATG TCGCTGAGG	TCGCAGTCGAG GGTCTCC	178	
SGPP1	+424	GGGAGCGACCGC AATCAG	CAGAACAGGCA GTAGAGCGG	191	
SGPP1	+485	AACTCGCTGACG GGCG	CAGAACAGGCA GTAGAGCGG	68	+319 to +652
SGPP1	+661	CATCTGGGTGCT GGTCATGTA	CCTCCAACTTGA CCACGGG	92	+507 to +816
SGPP1	+964	ATACTCGCAAACA GGCCCTT	GGCTAAGTATC CAGTTTCCCCC	68	+798 to +1131
SGPP1	+1108	TCATTGGGCTTTG GTTGAGTT	ACTTCCACTGTG CATTACAAGAA	60	+938 to +1279
SGPP1	+1168	TCATTGGGCTTTG GTTGAGTT	ACACTGTCTCAC GGTAAAGCTAA	180	
SGPP1	+1225	TGGTGCCTTCTTC CCTAAAACTGT	ACACTGTCTCAC GGTAAAGCTAA	67	+1058 to +1392
SGPP1	+1325	ACATGCTAGTTCC AAGCAATGAAG	CCCTGTGCTCC ATTAACTTCCAT A	74	+1162 to +1489
RUNX2	+66065	AAGGCCCCACCT CTAACACT	AGACAACAGGC GAGGCTAAA	115	
ID1	+275	GTGGCCATCTCG CGCT	AGCTCCTTGAG GCGTGAGTA	119	

Amplicon Positions relative to TSS determined for *SGPP1* from NC_000014.9 relative to nucleotide position 63,728,108. Amplicon positions relative to TSS for *ID1* [8] and *Runx2* [62]. *Detection ranges calculated: 5'end R -200-bp to 5'end F +200-bp

Table S5: Putative GFI1 DNA binding sites in the *SGPP1* gene detectable by the amplicons used in the 200-bp screen.

Type of site ID	bp relative to *SGPP1* TSS	Potentially detectable by the following amplicon(s) on 200-bp fragments *
Core	+210 - +213	+138
Core	+252 - +255	+138
Core/Jaspar/Transfac	+339 - +350	+485
Core	+356 - +359	+485
Core	+519 - +522	+485, **+661**
Jaspar	+872 - +883	**+964**
Core	+904 - +907	**+964**
Core/Jaspar/Transfac	+967 - +978	**+964, +1108**
Core	+1086 - +1089	**+964, +1108, +1225**
Core	+1109 - +1112	**+964, +1108, +1225**
Jasper	+1133 - +1144	**+1108, +1225**
Core	+1152 - +1155	**+1108, +1225**
Core	+1239 - +1242	**+1108, +1225,** +1325
Core	+1302 - +1305	**+1225,** +1325
Core	+1314 - +13317	**+1225,** +1325
Core	+1318 - +1321	**+1225,** +1325
Core	+1411 - +1414	+1325
Core	+1430 - +1433	+1325

*Amplicon midpoints from Table S4 and Figure S3. Bolded amplicons detected significant GFI1 binding in OPM2 and/or MM1S cells by GFI1-ChIP using 200-bp chromatin fragmentation (positive amplicons). Sites in a region detectable by both a positive amplicon and a negative amplicon are less likely to be functional GFI1 binding sites in MM cells.
